# Structural basis for recognition and transport of folic acid in mammalian cells

**DOI:** 10.1016/j.sbi.2022.102353

**Published:** 2022-03-15

**Authors:** Simon Newstead

**Affiliations:** 1 Department of Biochemistry, University of Oxford, Oxford, OX1 3QU, UK

## Abstract

Structural studies on mammalian vitamin transport lag behind other metabolites. Folates, also known as B9 vitamins, are essential cofactors in one-carbon transfer reactions in biology. Three different systems control folate uptake in the human body; folate receptors function to capture and internalise extracellular folates via endocytosis, whereas two major facilitator superfamily transporters, the reduced folate carrier (RFC; SLC19A1) and proton-coupled folate transporter (PCFT; SLC46A1) control the transport of folates across cellular membranes. Targeting specific folate transporters is being pursued as a route to developing new antifolates with improved pharmacology. Recent structures of the proton-coupled folate transporter, PCFT, revealed key insights into antifolate recognition and the mechanism of proton-coupled transport. Combined with previously determined structures of folate receptors and new predictions for the structure of the RFC, we are now able to develop a structure-based understanding of folate and antifolate recognition to accelerate efforts in antifolate drug development.

## Introduction

Solute carrier transporters play an important role in vitamin uptake in humans [1], yet our understanding of the biochemical and structural basis for their function lags that of equivalent transport systems. However, vitamin transporters represent excellent targets for developing novel anticancer medications [2], aiding our understanding of metabolic diseases [3], and treating inflammatory and cognitive disorders [4].

**Figure 1:**
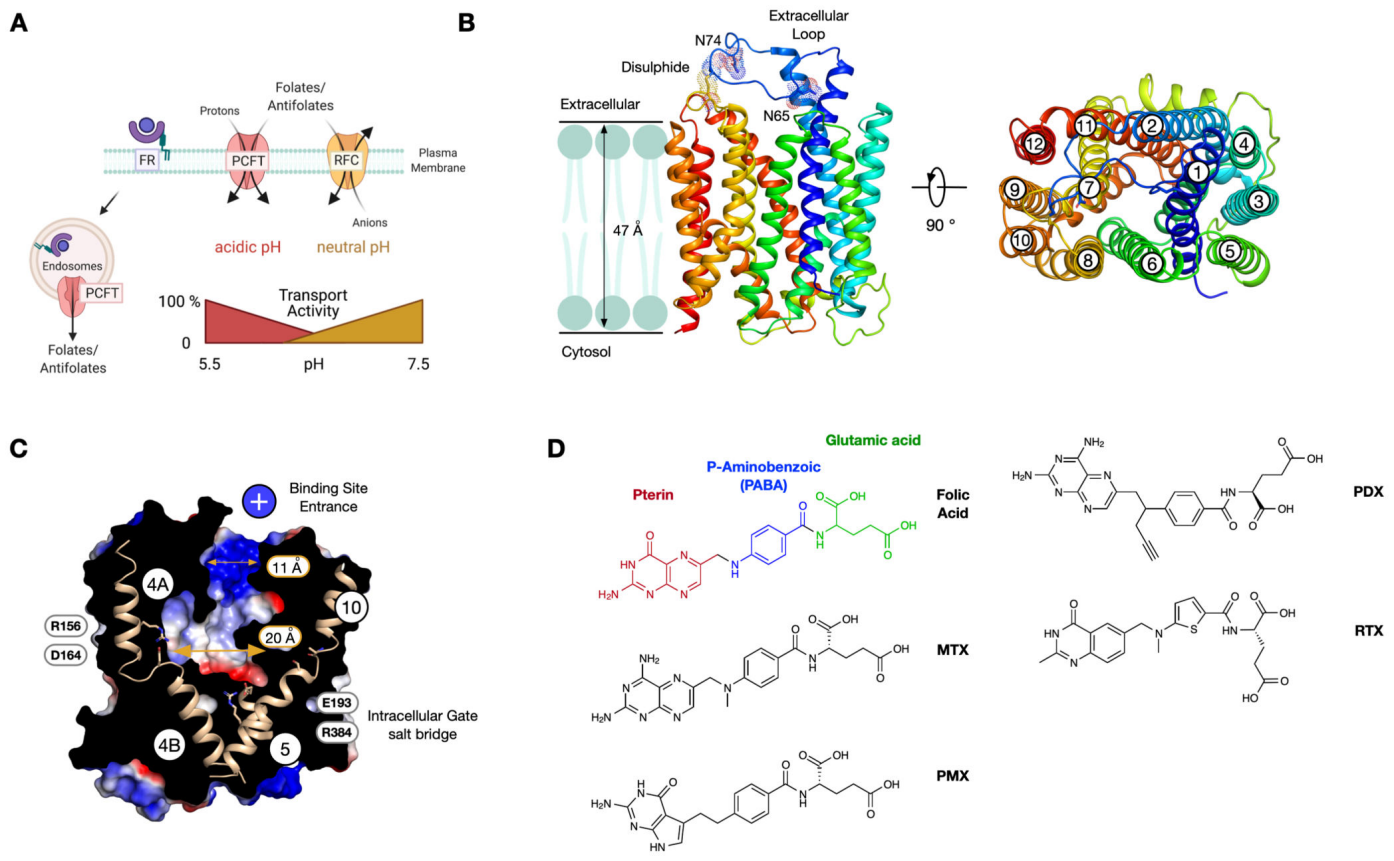
Folic acid transport systems and the structure of PCFT. **A.** Three biochemically distinct systems mediate folate transport in mammals and respond differently to changes in environmental pH. **B.** Cryo-EM structure of chicken PCFT (PDB: 7BC6) revealed an extracellular loop that connects the N- and C-terminal bundles on the extracellular side of the membrane via a disulphide bridge and the location of glycosylation sites. **C**. TM4 and TM10 contain partially unwound regions that function to lengthen the binding site relative to other MFS structures. Two conserved salt bridge interactions stabilise the outward open state. The binding site displays a noticeable constriction at the entrance which is positively charged. **D.** Chemical structure of Folic Acid and clinically used antifolates in the treatment of cancer and inflammatory diseases.

Folates, which comprise the family of B9 vitamins, function as cofactors in one-carbon transfer reactions and are required to synthesise nucleic acids, activated methyl groups for DNA methylation and the conversion of homocysteine to methionine [5,6]. In 2006, following the link between mutations in the *SLC46A1* gene and Hereditary Folate Malabsorption (HFM) syndrome, a metabolic disorder that results in severe developmental and neurological abnormalities [7,8], a new transporter for dietary folates, the proton-coupled folate transporter, PCFT (SLC46A1), was discovered [9]. PCFT belongs to the major facilitator superfamily (MFS) of secondary active transporters and is maximally active at pH 5.5, with transport dropping to negligible levels above pH 7.0 [10,11]. PCFT joins two previously identified folate uptake systems, the folate receptors (FRα & FRβ), which are glycosylphosphatidylinositol (GPI) anchored proteins that bind extracellular folates for internalization via endocytosis [12,13], and the reduced folate carrier (RFC; SLC19A1), also a member of the MFS, which operates at neutral to slightly alkaline pH, as a folate anion antiporter [14] (Fig. 1A).

Antifolates are an established treatment in cancer due to their ability to inhibit key enzymes in folate metabolism, including dihydrofolate reductase and thymidylate synthase, which lead to cell cycle arrest and apoptosis [15]. Toxicity associated with antifolates results from their transport into healthy cells, predominantly via RFC, which is widely expressed throughout the body and through the development of drug resistance [16,17]. In the acidic microenvironment of solid tumours, PCFT is more active than RFC [18] and thus presents an attractive opportunity to design more selective antifolates [15,19] through preferential uptake via PCFT. Such PCFT selective antifolates are under active development [20]. Similarly, folate receptors are upregulated in certain tumours, providing another route to selective uptake [21-23]. However, the structural basis by which these three biochemically distinct systems distinguish between antifolates remains unclear. Our current structure of the PCFT transporter [24], captured in both the ligand-free state and bound to pemetrexed, a second-generation antifolate, now provides an ideal opportunity to begin to understand folate transport via the SLC proteins and ligand specificity within and between these three distinct folate uptake systems.

## Structure of PCFT

The structure of chicken PCFT (PDB:7BC6) was recently obtained at 3.2 Å resolution through single particle Cryo-EM [24]. The structure revealed a 12 transmembrane helical protein, displaying the canonical MFS fold consisting of two six-helix bundles that pack together in the plane of the membrane to form a ‘V’-shaped molecule [25] (Fig. 1B). Two unique structural features were immediately apparent. The first was a large extracellular loop between TM1 and TM2, which reaches across the extracellular part of the transporter and forms a disulphide bond with the peptide chain connecting TM7 and TM8 [26]. The loop functions to structurally connect the transporter’s N- and C-terminal halves on the extracellular side of the membrane. Although the disulphide bond is not required for transport activity [24,26], the extracellular loop contains two conserved N-glycosylation sites at position Asn65 and Asn74 (Asn58 & Asn68 in human PCFT) [27], which may be important for trafficking or quality control *in vivo*. The second feature is the presence of a large discontinuity within TM4, which is stabilized through an intrahelical salt bridge between Arg156 and Asp164 (Arg148 & Asp156 in human PCFT) (Fig. 1C).

The discontinuity breaks TM4 into two half helices TM4A (extracellular half) and TM4B (cytoplasmic half). TM4A is translated ~ 5.5 Å away from TM4B, creating an extended pocket within the N-terminal bundle. Interestingly, a similar but much smaller discontinuity occurs in TM10, the symmetry-related helix in the C-terminal bundle. The position of the extracellular loop results in the close positioning of TM1 and TM7 at the mouth of the binding site, resulting in a narrow entrance relative to the bottom of the binding site, ~ 11 Å vs ~ 20 Å. This narrow entrance, coupled with the translation in TM4A and TM10, results in the outward open transporter’s ‘bottle neck vase’ structure (Fig. 1C). The entrance vestibule is also positively charged, which would attract negatively charged folates. Finally, the cytoplasmic side of the transporter is sealed through the packing together of TMs 4-5 with TMs 10-11 and stabilized through a second conserved salt bridge between Glu193 on TM5 and Arg384 on TM10 (Fig.1C).

## Mechanisms of folate and antifolate recognition

Classical and rationally designed antifolates continue to play a key role in cancer therapy in addition to inflammatory diseases such as rheumatoid arthritis and psoriasis [20,28]. Clinically important antifolates include methotrexate (MTX), pemetrexed (PMX), pralatrexate (PDX) and raltitrexed (RTX), which function as competitive inhibitors for different enzymes in folate metabolism [17] (Fig. 1D). All are excellent substrates for RFC, FRs and PCFT and differ from folic acid through either substitution on the pterin ring combined with modifications of the carbon bridge to the p-aminobenzoic acid (PABA) group (MTX, PDX) or replacing the p-aminobenzoic acid group with an alternative ring, such as a thiophene (RTX) or replacing the pterin ring with a pyrrolo-pyrimidine analogue (PMX). However, the transport of antifolates by RFC confers limited tumour selectivity during chemotherapy [29], prompting the development of antifolates targeted to either PCFT or the folate receptors to improve efficacy and reduce side effect. Pemetrexed (Alimta) was approved for the treatment of malignant pleural mesothelioma in 2004 and for metastatic non-small cell lung cancer in combination with cisplatin in 2008 [30,31]. Within currently administered antifolates PMX displays the lowest KM of 0.2 - 0.8 μM and IC50 of 0.2 μM for PCFT [32,33] compared to RFC (1.1 μM) [34], and is currently being exploited to develop more targeted antifolates [19,35-37]. The structure of chicken PCFT in complex with PMX was also obtained at 3.3 Å resolution (PDB:7BC7), joining a previously reported structure of the human FRβ in complex with PMX at 2.6 Å (PDB:4KN2) [38], enabling a direct comparison of the structural features for PMX recognition between the transporter and receptor systems.

**Figure 2:**
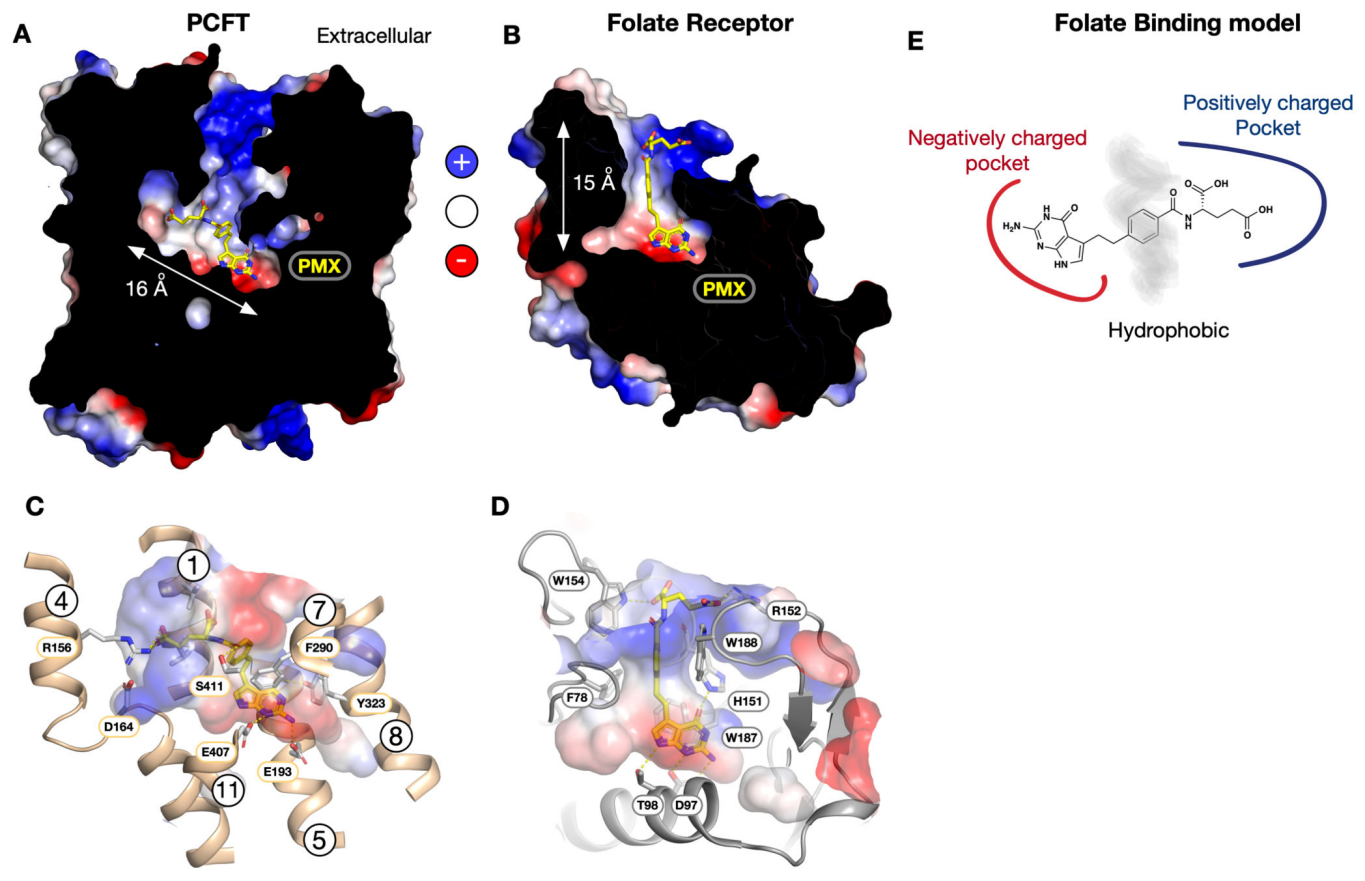
Structural comparison between PCFT and Folate Receptor binding sites. **A.** Cryo-EM structure of chicken PCFT bound to pemetrexed (PMX) (PDB: 7BC7). The electrostatic surface displays a pronounced charge distribution in the folate binding site. **B.** Crystal structure of the human folate receptor beta bound to pemetrexed (PDB:4KN2), which displays a similar electrostatic distribution in the folate binding site to PCFT. **C.** Zoomed in view of the folate binding site displaying the bound PMX, key interacting side chains and the electrostatic surface. **D.** Similar zoomed in view of the FRβ folate binding site. **E.** Schematic binding model highlighting the importance of electrostatics in folate binding.

Interestingly the binding site architecture in PCFT and FRβ is relatively similar in that both have a ‘bottleneck vase’ structure (Fig. 2A & B). However, whereas pemetrexed is bound horizontally in PCFT, connecting with side chains in both the N- and C-terminal bundles, in FRβ it adopts a vertical orientation, with the pyrrolo-pyrimidine group in the base of the cavity and the glutamate group projecting out towards the cytoplasmic space [38]. We also observe similarities in the electrostatics, with both sites coordinating the γ-carboxylate group of the glutamate with arginine (Arg156 on TM4 in PCFT & Arg152 in FRβ). In contrast, the pyrrolo-pyrimidine is coordinated by negatively charged side chains in both binding sites (Glu193 and Glu407 on TMs 5 and 11 in PCFT & Asp97 in hFRβ) (Fig. 2C & D). The exocyclic oxygen on the pyrrolo-pyrimidine group makes interactions in both binding sites (Tyr323 on TM8 in chicken PCFT and His151 in human FRβ). Additional interactions are made to the nitrogen of the pyrrolo group by a threonine in FRβ (Thr98). The PABA group is accommodated in both proteins in a hydrophobic band between the two opposing charged regions. Interestingly, a similar arrangement of charged groups is observed in the binding site in B12-dependent methionine synthase (MetH) [39] and the energy coupling factor transporter FolT [40,41], possibly suggesting a convergent mechanism for folate recognition. The ring groups are accommodated in negatively charged pockets containing H-bond acceptors and donors. The PABA and bridging group variants are accommodated in a hydrophobic region, characterized by the presence of aromatics, whereas the opposite end of the ligand is accommodated in a positively charged region, dominated by the presence of arginine side chains that form salt bridges with either the α or γ carboxylate of the glutamate group. Taken together, the analysis of PCFT and FRβ suggest that folic acid binding sites display conserved features in these proteins (Fig. 2E).

## A transport mechanism for PCFT

**Figure 3:**
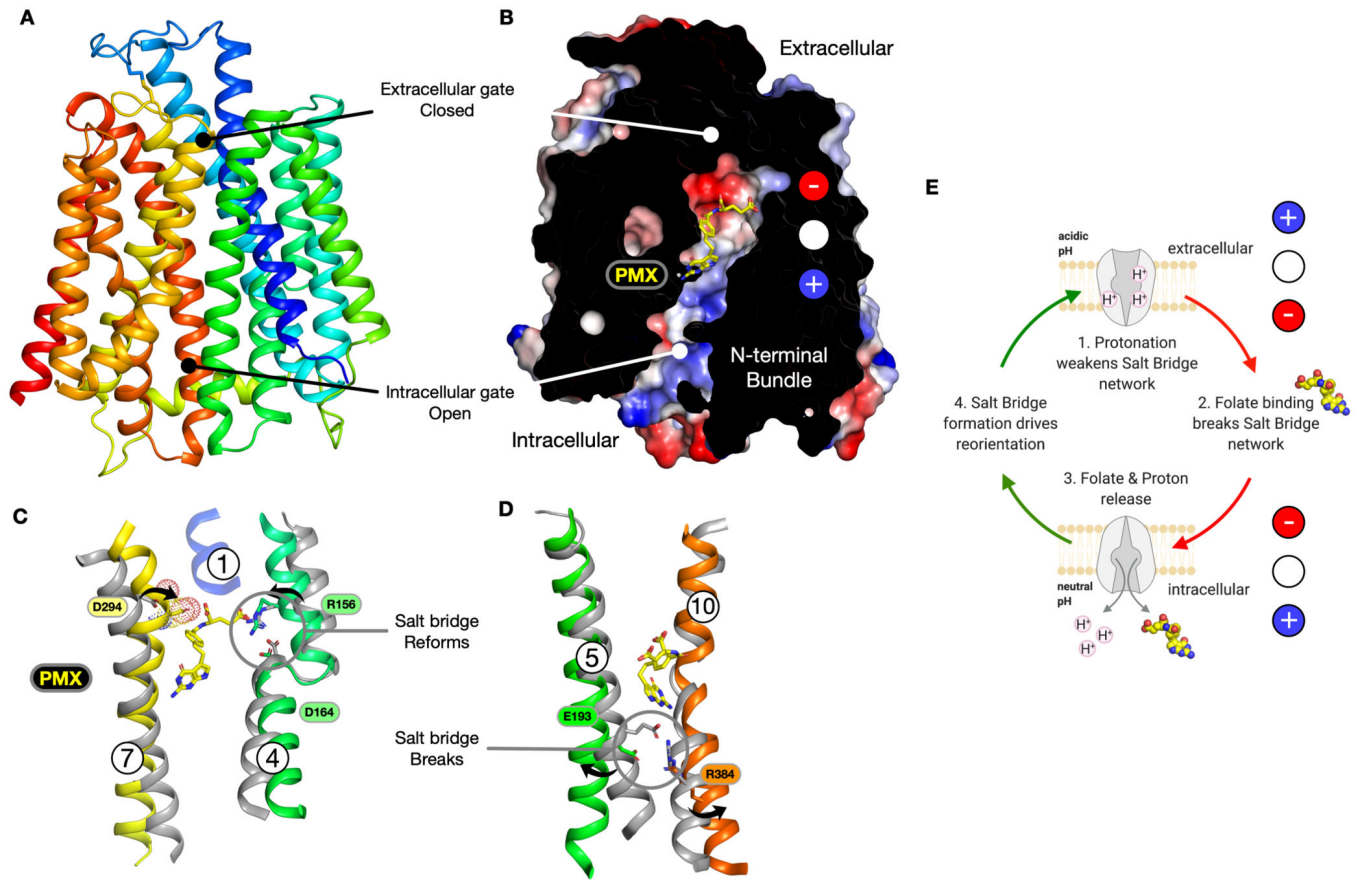
Electrostatics play a key role in transport by PCFT. **A.** AlphaFold (AF) predicted model of the human PCFT (SLC46A1) protein in the inward open conformation (coloured from the N-terminus blue to C-terminus red). The extracellular and intracellular gates are labelled. **B.** Equivalent view for the human PCFT AF model showing the electrostatic surface charges. The antifolate PMX has been modelled in by superimposing the chicken PCFT structure (PDB: 7BC7) and transferring the coordinates for PMX – no further relaxing of the PMX structure or protein was conducted. Note the electrostatic charge distribution in the binding site is reversed relative to that in outward open state. **C & D.** Structural overlays between the outward open structure (Grey; PDB: 7BC7) and inward open AF model for human PCFT (coloured as in **A**) for the extracellular and intracellular gate helices respectively. **E.** Model for proton coupled transport by PCFT as originally proposed in [24] but including the predicted charge distribution changes in the ligand binding site.

Alternating access transport within the MFS can be understood in terms of gates, which control access to a central binding site within the transporter [42]. In the outward open state of the transport cycle, the intracellular gate, which is constructed by TMs 4-5 packing against TMs 10-11 is closed, whereas the extracellular gate, constructed by TMs 1-2 and TMs 7-8 is open [43]. PCFT contains two conserved salt bridge interactions that stabilise the outward-facing state, namely the intrahelical salt bridge in TM4 (Arg156-Asp164) and Glu193 and Arg384, which stabilises the packing of TM5 with TM10 (Fig. 1C) [24]. However, at present, we only have one state for PCFT, leaving questions around the transition to the inward open state and release of folate and protons to the cytoplasm unanswered. However, we can use the predicted AlphaFold model of the inward open human PCFT protein to analyse the structural movements that might occur during proton-coupled transport (Fig. 3A).

Overlaying the Cryo-EM structure of PMX bound outward open chicken PCFT onto the inward open AlphaFold model of the human protein suggests the electrostatic environment has changed dramatically (Fig. 3B). Indeed, it appears to have reversed, with the positively charged pocket holding the glutamate in the outward open state becoming negative in the inward open state. Similarly, at the other end of the ligand the pyrrolo-pyrimidine binding pocket changes from negative in the outward open state to slightly positive in the inward open conformation. This switch occurs following the movement of the gating helices. TM7 moves inwards to pack against TM1, which moves a conserved aspartate Asp294 (Asp286 in human PCFT) into the binding pocket and towards α-carboxyl on PMX (Fig. 3C). The proximity of the aspartate to the α-carboxyl group will cause a repulsive interaction, facilitating the unbinding of PMX. Following the opening of the binding site to the cytoplasm, the proton bound to Asp164 will also depart. This will enable the salt bridge on TM4 to reengage, simultaneously removing the interaction between Arg156 to the γ-carboxylate and reducing the positive electrostatic charge in the glutamate pocket, further facilitating ligand release. Movement of TMs1-2 and TMs 7-8 also results in opening the intracellular gate. Consistent with this movement, we observe the salt bridge between Glu193 on TM5 and Arg384 on TM10 is broken in the inward open predicted model (Fig. 3D). The breaking of this salt bridge is likely facilitated by protonation of Glu193, as predicted [24]. Adopting the inward open state would also destroy the interactions that hold the pyrrolo-pyrimidine in the outward open state and opens a pathway to the cytoplasm. This co-occurs with the development of the negative charge near the α-carboxyl, discussed above, which would provide an additional force to promote the release of PMX into the cell.

Our mechanism thus contains a concurrent switch in the formation and breakage of the conserved salt bridge pairs, elaborating the mechanism proposed in [24] (Fig. 3E). In the outward open state, the TM4 salt bridge is broken following protonation of Asp164 from the extracellular side of the membrane, freeing Arg156 to bind the γ-carboxylate of folic acid (or antifolate drugs). In contrast, the TM5-TM10 salt bridge remains intact. However, additional protonation of Glu193 on TM5 weakens this interaction, resulting in the opening of the intracellular gate following ligand binding and interaction with the exocyclic amine group on the pyrrolo-pyrimidine ring. In the inward open state, the TM4 salt bridge reforms, whilst the TM5-TM10 salt bridge is broken. The transporter can then reset to the outward open state following the reformation of the TM5-TM10 salt bridge, which presumably facilitates the opening of the extracellular gate. Concurrent with these structural changes, the binding site likely also undergoes a dramatic shift in the electrostatic character, which facilitates folate binding from the extracellular side and release on the intracellular side of the membrane. In the above model, only two protons are thermodynamically coupled to folate transport. However, human PCFT is electrogenic [9], suggesting that in humans, at least three or more protons are transported per folate (folic acid has a -2 charge). It is unclear whether these additional protons are thermodynamically coupled or facilitate transport kinetically, possibly via a conserved histidine [24]. Further biochemical studies will be needed to address this question.

## The Reduced Folate Carrier

**Figure 4:**
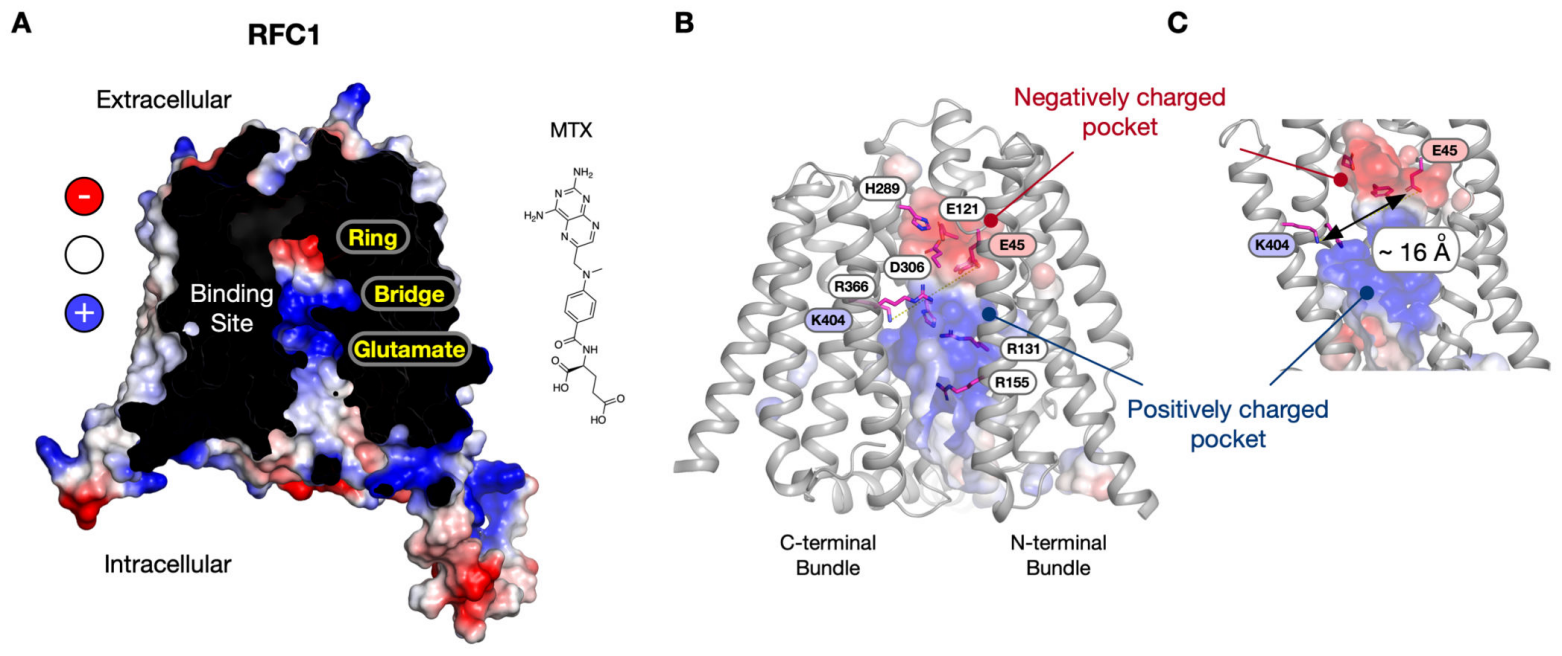
AlphaFold model for the structure of the Reduced Folate Carrier. **A.** The AlphaFold model for mouse RFC (SLC19A1) predicts an inward open state that displays a similar pronounced electrostatic distribution in the central cavity of the transporter. Inset – chemical structure of the antifolate methotrexate (MTX). **B.** Cartoon representation of the RFC model highlighting the central binding site and the location of functionally important side chains, including Glu45 side chain in the acidic pocket and Lys404 in the basic pocket. **C.** Zoomed in view of the binding site highlighting the similar distance between two key side chains that have been implicated in binding the glutamate and pterin rings of MTX.

The recent release of accurate structure predictions using the EBI server (https://alphafold.ebi.ac.uk/) coupled with AlphaFold [44] provides an opportunity to compare the calculated structure of RFC with our experimentally available models of chicken PCFT, and human FRβ discussed above. Interestingly, the predicted RFC structure from *Mus musculus*, which was modelled in the inward open conformation, also contains a very pronounced electrostatic dipole in the binding site (Fig. 4A). (The predicted human structure contained structural irregulates and was not analysed here). In the AF predicted mouse RFC model, the negative electrostatic patch is at the extracellular end of the binding site and the positive electrostatic region at the intracellular side. Similar to PCFT and FRβ, we observe a hydrophobic band separating the ring binding pocket and the glutamate recognition site, consistent with our binding model (Fig. 2E). Suppose we apply our logic from the binding model discussed above for PCFT and FRβ to the predicted structure of RFC. In that case, we might expect antifolates to orientate in the binding site with their ring groups positioned towards the extracellular gate, and the glutamate group towards the cytoplasm (Fig. 4A). A glutamate to a lysine mutation at amino acid 45 in the human RFC transporter (Glu45Lys on TM1) was identified in a MTX resistant cell line, which also showed decreased activity towards 5-formyl-tetrahydrofolate (5-CHO-THF) [45]. Glutamate 45 is located within the negatively charged acidic region, consistent with our predicted model (Fig. 4B). A lysine substitution in this region would be expected to negatively impact interactions with the pterin ring in MTX and with THF (tetrahydrofolate).

Similarly, in the positively charged patch of the binding site, a conserved lysine on TM11 (Lys404 in mouse RFC; Lys411 in human RFC) was found to interact with the γ-carboxyl group on the glutamate moiety [46], consistent with the proposed orientation of MTX in Fig 4A. Intriguingly, these two oppositely charged groups are positioned ~ 16 Å apart (Fig. 4C), consistent with the distances observed in PCFT and FRβ (Fig. 2A & B). RFC also contains several conserved arginine side chains within the central binding site that are essential for function [47] and align with this positively charged region. Although confirmation of this structural prediction awaits experimental verification, the current ligand binding model, involving charge separation between basic and acidic regions via a hydrophobic gap, is consistent with the current available mutational and functional data [48].

## Future perspective

Although further structures and drug complexes are needed to form a complete picture of the transport cycle, we can start rationalising current antifolate selectivity data [19,49,50]. Indeed, the current structure of PCFT in complex with PMX already rationalised a minimal length requirement for ligands to trigger transport, consistent with the need to disrupt the two conserved salt bridge interactions in the binding site [24]. This prior information can now be combined with the insights highlighted here on the role of electrostatics, how these are likely to influence ligand capture and release and the different binding orientations between the folate transport systems. However, an important consideration will be the effect of the external pH environment on ligand recognition, particularly concerning RFC and PCFT, which function at very different pH optima [18]. Further biochemical studies on these systems will illuminate the impact pH has on binding affinities and kinetics. Nevertheless, the emerging insights into folate and antifolate binding within the three systems will accelerate efforts to develop more selective antifolates for improved cancer treatment.

## References

[1] Litwack G (2021). Human biochemistry.

[2] Papalazarou V, Maddocks ODK (2021). Supply and demand: Cellular nutrient uptake and exchange in cancer. Mol Cell.

[3] Schumann T, Konig J, Henke C, Willmes DM, Bornstein SR, Jordan J, Fromm MF, Birkenfeld AL (2020). Solute Carrier Transporters as Potential Targets for the Treatment of Metabolic Disease. Pharmacol Rev.

[4] Al-Abdulla R, Perez-Silva L, Abete L, Romero MR, Briz O, Marin JJG (2019). Unraveling 'The Cancer Genome Atlas' information on the role of SLC transporters in anticancer drug uptake. Expert Rev Clin Pharmacol.

[5] Bailey LB (2010). Folate in health and disease.

[6] Lan X, Field MS, Stover PJ (2018). Cell cycle regulation of folate-mediated one-carbon metabolism. Wiley Interdiscip Rev Syst Biol Med.

[7] Kronn D, Goldman ID, Adam MP, Ardinger HH, Pagon RA, Wallace SE, Bean LJH, Stephens K, Amemiya A (1993). GeneReviews((R)).

[8] Salojin KV, Cabrera RM, Sun W, Chang WC, Lin C, Duncan L, Platt KA, Read R, Vogel P, Liu Q (2011). A mouse model of hereditary folate malabsorption: deletion of the PCFT gene leads to systemic folate deficiency. Blood.

[9] Qiu A, Jansen M, Sakaris A, Min SH, Chattopadhyay S, Tsai E, Sandoval C, Zhao R, Akabas MH, Goldman ID (2006). Identification of an intestinal folate transporter and the molecular basis for hereditary folate malabsorption. Cell.

[10] Nakai Y, Inoue K, Abe N, Hatakeyama M, Ohta KY, Otagiri M, Hayashi Y, Yuasa H (2007). Functional characterization of human proton-coupled folate transporter/heme carrier protein heterologously expressed in mammalian cells as a folate transporter. J Pharmacol Exp Ther.

[11] Qiu A, Min SH, Jansen M, Malhotra U, Tsai E, Cabelof DC, Matherly LH, Zhao R, Akabas MH, Goldman ID (2007). Rodent intestinal folate transporters (SLC46A1) secondary structure, functional properties, and response to dietary folate restriction. Am J Physiol Cell Physiol.

[12] Kamen BA, Smith AK (2004). A review of folate receptor alpha cycling and 5-methyltetrahydrofolate accumulation with an emphasis on cell models in vitro. Adv Drug Deliv Rev.

[13] Kamen BA, Wang MT, Streckfuss AJ, Peryea X, Anderson RG (1988). Delivery of folates to the cytoplasm of MA104 cells is mediated by a surface membrane receptor that recycles. J Biol Chem.

[14] Matherly LH, Hou Z (2008). Structure and function of the reduced folate carrier a paradigm of a major facilitator superfamily mammalian nutrient transporter. Vitam Horm.

[15] Goldman ID, Chattopadhyay S, Zhao R, Moran R (2010). The antifolates: evolution, new agents in the clinic, and how targeting delivery via specific membrane transporters is driving the development of a next generation of folate analogs. Curr Opin Investig Drugs.

[16] Zhao R, Goldman ID (2003). Resistance to antifolates. Oncogene.

[17] Gonen N, Assaraf YG (2012). Antifolates in cancer therapy: structure, activity and mechanisms of drug resistance. Drug Resist Updat.

[18] Visentin M, Diop-Bove N, Zhao R, Goldman ID (2014). The intestinal absorption of folates. Annu Rev Physiol.

[19] Matherly LH, Hou Z, Gangjee A (2018). The promise and challenges of exploiting the proton-coupled folate transporter for selective therapeutic targeting of cancer. Cancer Chemother Pharmacol.

[20] Visentin M, Zhao R, Goldman ID (2012). The antifolates. Hematol Oncol Clin North Am.

[21] Gibbs DD, Theti DS, Wood N, Green M, Raynaud F, Valenti M, Forster MD, Mitchell F, Bavetsias V, Henderson E (2005). BGC 945, a novel tumor-selective thymidylate synthase inhibitor targeted to alpha-folate receptor-overexpressing tumors. Cancer Res.

[22] Matherly LH, Goldman DI (2003). Membrane transport of folates. Vitam Horm.

[23] Ross JF, Chaudhuri PK, Ratnam M (1994). Differential regulation of folate receptor isoforms in normal and malignant tissues in vivo and in established cell lines. Physiologic and clinical implications Cancer.

[24] Parker JL, Deme JC, Kuteyi G, Wu Z, Huo J, Goldman ID, Owens RJ, Biggin PC, Lea SM, Newstead S (2021). Structural basis of antifolate recognition and transport by PCFT. Nature.

[25] Yan N (2015). Structural Biology of the Major Facilitator Superfamily Transporters. Annu Rev Biophys.

[26] Zhao R, Unal ES, Shin DS, Goldman ID (2010). Membrane Topological Analysis of the Proton-Coupled Folate Transporter (PCFT-SLC46A1) by the Substituted Cysteine Accessibility Method. Biochemistry.

[27] Unal ES, Zhao R, Qiu A, Goldman ID (2008). N-linked glycosylation and its impact on the electrophoretic mobility and function of the human proton-coupled folate transporter (HsPCFT). Biochim Biophys Acta.

[28] Shinde CG, Venkatesh MP, Kumar TM, Shivakumar HG (2014). Methotrexate: a gold standard for treatment of rheumatoid arthritis. J Pain Palliat Care Pharmacother.

[29] Desmoulin SK, Hou Z, Gangjee A, Matherly LH (2012). The human proton-coupled folate transporter: Biology and therapeutic applications to cancer. Cancer Biol Ther.

[30] Cohen MH, Cortazar P, Justice R, Pazdur R (2010). Approval summary: pemetrexed maintenance therapy of advanced/metastatic nonsquamous, non-small cell lung cancer (NSCLC). Oncologist.

[31] Hazarika M, White RM, Johnson JR, Pazdur R (2004). FDA drug approval summaries: pemetrexed (Alimta). Oncologist.

[32] Diop-Bove NK, Wu J, Zhao R, Locker J, Goldman ID (2009). Hypermethylation of the human proton-coupled folate transporter (SLC46A1) minimal transcriptional regulatory region in an antifolate-resistant HeLa cell line. Mol Cancer Ther.

[33] Zhao R, Qiu A, Tsai E, Jansen M, Akabas MH, Goldman ID (2008). The proton-coupled folate transporter: impact on pemetrexed transport and on antifolates activities compared with the reduced folate carrier. Mol Pharmacol.

[34] Wang Y, Zhao R, Goldman ID (2004). Characterization of a folate transporter in HeLa cells with a low pH optimum and high affinity for pemetrexed distinct from the reduced folate carrier. Clin Cancer Res.

[35] Golani LK, Wallace-Povirk A, Deis SM, Wong J, Ke J, Gu X, Raghavan S, Wilson MR, Li X, Polin L (2016). Tumor Targeting with Novel 6-Substituted Pyrrolo [2,3-d] Pyrimidine Antifolates with Heteroatom Bridge Substitutions via Cellular Uptake by Folate Receptor alpha and the Proton-Coupled Folate Transporter and Inhibition of de Novo Purine Nucleotide Biosynthesis. J Med Chem.

[36] Golani LK, Islam F, O'Connor C, Dekhne AS, Hou Z, Matherly LH, Gangjee A (2020). Design, synthesis and biological evaluation of novel pyrrolo[2,3-d]pyrimidine as tumor-targeting agents with selectivity for tumor uptake by high affinity folate receptors over the reduced folate carrier. Bioorg Med Chem.

[37] Wang L, Cherian C, Kugel Desmoulin S, Mitchell-Ryan S, Hou Z, Matherly LH, Gangjee A (2012). Synthesis and biological activity of 6-substituted pyrrolo[2,3-d]pyrimidine thienoyl regioisomers as inhibitors of de novo purine biosynthesis with selectivity for cellular uptake by high affinity folate receptors and the proton-coupled folate transporter over the reduced folate carrier. J Med Chem.

[38] Wibowo AS, Singh M, Reeder KM, Carter JJ, Kovach AR, Meng W, Ratnam M, Zhang F, Dann CE (2013). Structures of human folate receptors reveal biological trafficking states and diversity in folate and antifolate recognition. Proc Natl Acad Sci U S A.

[39] Evans JC, Huddler DP, Hilgers MT, Romanchuk G, Matthews RG, Ludwig ML (2004). Structures of the N-terminal modules imply large domain motions during catalysis by methionine synthase. Proc Natl Acad Sci U S A.

[40] Swier LJ, Guskov A, Slotboom DJ (2016). Structural insight in the toppling mechanism of an energy-coupling factor transporter. Nat Commun.

[41] Zhao Q, Wang C, Wang C, Guo H, Bao Z, Zhang M, Zhang P (2015). Structures of FolT in substrate-bound and substrate-released conformations reveal a gating mechanism for ECF transporters. Nat Commun.

[42] Drew D, Boudker O (2016). Shared Molecular Mechanisms of Membrane Transporters. Annu Rev Biochem.

[43] Fowler PW, Orwick-Rydmark M, Radestock S, Solcan N, Dijkman PM, Lyons JA, Kwok J, Caffrey M, Watts A, Forrest LR (2015). Gating topology of the proton-coupled oligopeptide symporters. Structure.

[44] Jumper J, Evans R, Pritzel A, Green T, Figurnov M, Ronneberger O, Tunyasuvunakool K, Bates R, Zidek A, Potapenko A (2021). Highly accurate protein structure prediction with AlphaFold. Nature.

[45] Zhao R, Assaraf YG, Goldman ID (1998). A mutated murine reduced folate carrier (RFC1) with increased affinity for folic acid, decreased affinity for methotrexate, and an obligatory anion requirement for transport function. J Biol Chem.

[46] Deng Y, Hou Z, Wang L, Cherian C, Wu J, Gangjee A, Matherly LH (2008). Role of lysine 411 in substrate carboxyl group binding to the human reduced folate carrier, as determined by site-directed mutagenesis and affinity inhibition. Mol Pharmacol.

[47] Sharina IG, Zhao R, Wang Y, Babani S, Goldman ID (2001). Mutational analysis of the functional role of conserved arginine and lysine residues in transmembrane domains of the murine reduced folate carrier. Mol Pharmacol.

[48] Hou Z, Matherly LH (2014). Biology of the major facilitative folate transporters SLC19A1 and SLC46A1. Curr Top Membr.

[49] Desmoulin SK, Wang Y, Wu J, Stout M, Hou Z, Fulterer A, Chang MH, Romero MF, Cherian C, Gangjee A (2010). Targeting the proton-coupled folate transporter for selective delivery of 6-substituted pyrrolo[2,3-d]pyrimidine antifolate inhibitors of de novo purine biosynthesis in the chemotherapy of solid tumors. Mol Pharmacol.

[50] Wang L, Cherian C, Desmoulin SK, Polin L, Deng Y, Wu J, Hou Z, White K, Kushner J, Matherly LH (2010). Synthesis and antitumor activity of a novel series of 6-substituted pyrrolo[2,3-d]pyrimidine thienoyl antifolate inhibitors of purine biosynthesis with selectivity for high affinity folate receptors and the proton-coupled folate transporter over the reduced folate carrier for cellular entry. J Med Chem.

